# Supplementary material for: Allyl Isothiocianate Induces Ca2+ Signals and Nitric Oxide Release by Inducing Reactive Oxygen Species Production in the Human Cerebrovascular Endothelial Cell Line hCMEC/D3
Source: Cells. 2023 Jun 27;12(13):1732. doi: 10.3390/cells12131732 (PMC10340171; doi:10.3390/cells12131732)
Supplement: Supplementary file 1 [file cells-12-01732-s001.zip › cells-2449117-supplementary.pdf]

Supplementary Figures

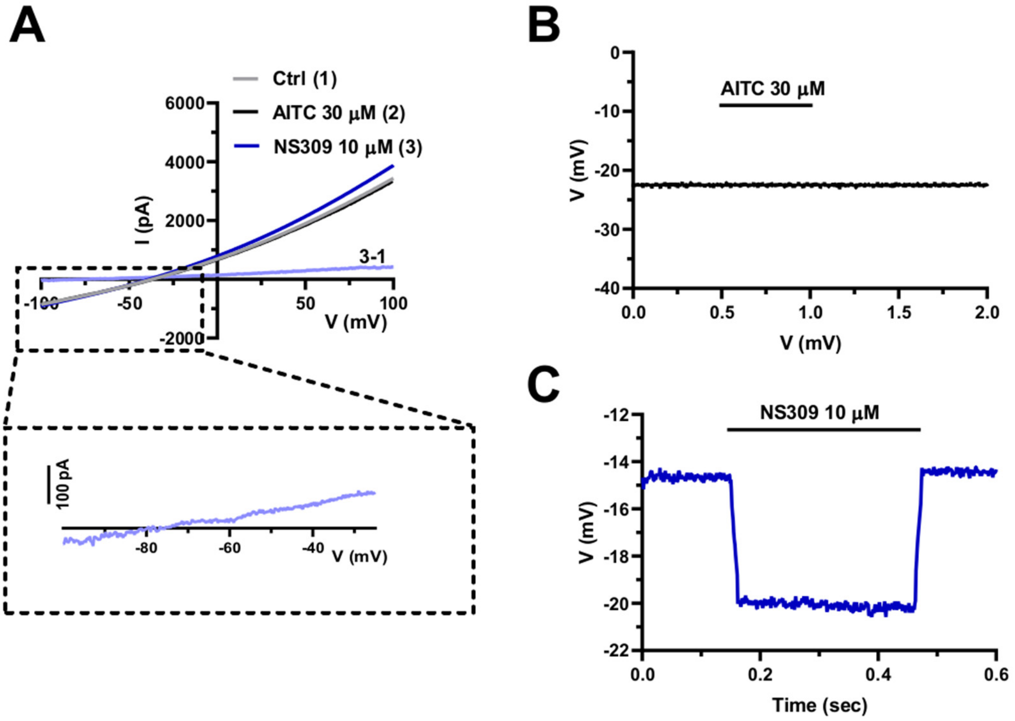

Figure S1. AITC does not induce bioelectrical signals in hCMEC/D3 cells. A, planar whole-cell patch-clamp recordings showed that AITC (30 μM) failed to activate transmembrane currents, while NS309 (10 μM), a selective opener of SK_{Ca}/IK_{Ca} channels, evoked an outwardly-rectifying K^+ current that reversed at around -70 mV, as shown in the inset. B, current-clamp recordings confirmed that AITC (30 μM) did not change the resting membrane potential, which ranges between -50 mV and -10 mV, as reported in other endothelial cell types (Moccia et al., 2002). C, NS309 (10 μM) induced a rapid and reversible membrane hyperpolarization, as predicted by the opening of SK_{Ca}/IK_{Ca} channels.

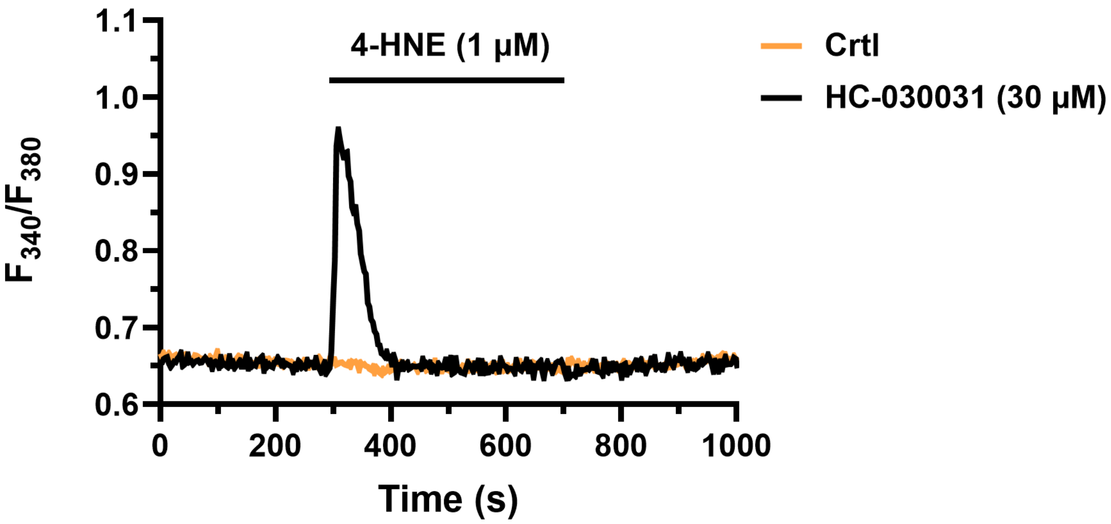

Figure S2. 4-HNE evokes TRPA1-mediated Ca^{2+} signals in hCMEC/D3 cells. 4-HNE (1 μM), a selective TRPA1 agonist, evoked a rapid increase in $[Ca^{2+}]_i$ in hCMEC/D3 cells (n=73 from three independent experiments). Conversely, 4-HNE failed to induce any detectable Ca^{2+} signal in the presence of HC-030031 (30 μM) (n=45 from three independent experiments).

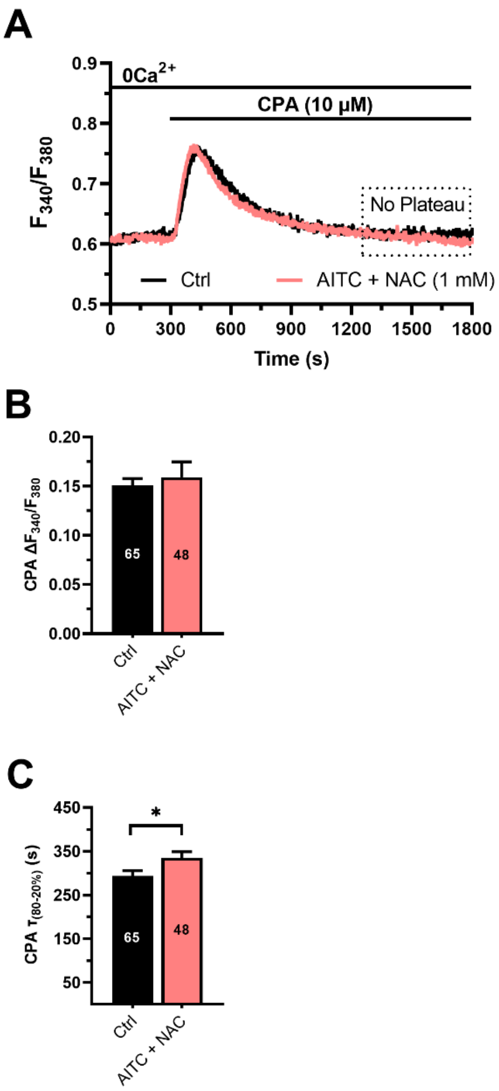

Figure S3. NAC prevents AITC effect on CPA-evoked Ca^{2+} release in hCMEC/D3 cells. A, pretreating hCMEC/D3 cells with the antioxidant NAC (1 mM) prevents AITC (30 µM) from converting CPA-evoked transient Ca^{2+} release into a long-lasting elevation in $[Ca^{2+}]_i$ presenting a discernible Ca^{2+} plateau. CPA was administered at 10 µM. Ctrl: control. B, mean±SE of the amplitude of CPA-evoked ER Ca^{2+} release in the absence (Ctrl) and presence of AITC+NAC. NS: not significant, Student's *t*-test. C, mean±SE of $\tau_{80\text{-}20}$ of CPA-evoked ER Ca^{2+} release in the absence (Ctrl) and presence of AITC+NAC. Student's *t*-test: * $p < 0.05$.

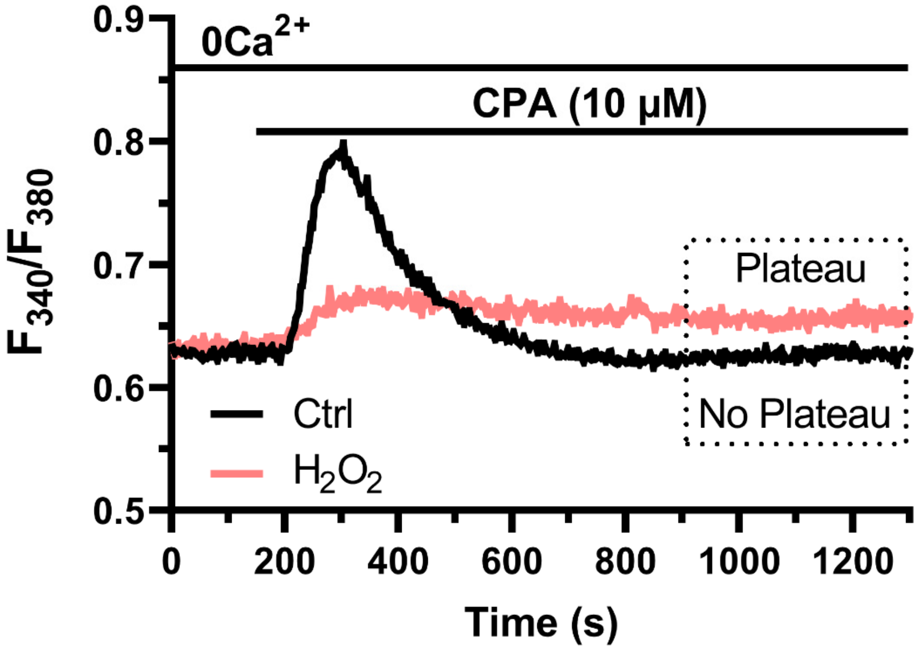

Figure S4. H_2O_2 prevents the decay to the baseline of CPA-evoked Ca^{2+} release. A, pretreating hCMEC/D3 cells with H_2O_2 (100 µM) reduces the amplitude of CPA-induced ER Ca^{2+} release from 0.1533± 0.00702 a.u. (n=42) measured under control (Ctrl) conditions to 0.06147± 0.004823 a.u. (n=42) (H_2O_2) (Student's *t*-test: **** $p < 0.0001$). The reduction in the peak amplitude of CPA-induced ER Ca^{2+} release is due to the reduction in the driving-force promoting intraluminal Ca^{2+} efflux because of the previous Ca^{2+} response to H_2O_2 (not shown). Nevertheless, in the presence of H_2O_2, the rise in $[Ca^{2+}]_i$ induced by CPA failed to decay to the baseline, resulting in a long-lasting plateau phase (0.01584± 0.003552 a.u., n=42). The baseline of the Ca^{2+} tracings shown in the Figure have been overlapped for representative purposes.

Reference

Moccia, F., Berra-Romani, R., Baruffi, S., Spaggiari, S., Adams, D.J., Taglietti, V., et al. (2002). Basal nonselective cation permeability in rat cardiac microvascular endothelial cells. *Microvasc Res* 64(2)**,** 187-197.
